# Machine learning classifiers provide insight into the relationship between microbial communities and bacterial vaginosis

**DOI:** 10.1186/s13040-015-0055-3

**Published:** 2015-08-12

**Authors:** Daniel Beck, James A. Foster

**Affiliations:** Department of Biological Sciences and Institute for Bioinformatics and Evolutionary Studies, University of Idaho, Moscow, ID USA

## Abstract

**Background:**

Bacterial vaginosis (BV) is a disease associated with the vagina microbiome. It is highly prevalent and is characterized by symptoms including odor, discharge and irritation. No single microbe has been found to cause BV. In this paper we use random forests and logistic regression classifiers to model the relationship between the microbial community and BV. We use subsets of the microbial community features in order to determine which features are important to the classification models.

**Results:**

We find that models generated using logistic regression and random forests perform nearly identically and identify largely similar important features. Only a few features are necessary to obtain high BV classification accuracy. Additionally, there appears to be substantial redundancy between the microbial community features.

**Conclusions:**

These results are in contrast to a previous study in which the important features identified by the classifiers were dissimilar. This difference appears to be the result of using different feature importance measures. It is not clear whether machine learning classifiers are capturing patterns different from simple correlations.

**Electronic supplementary material:**

The online version of this article (doi:10.1186/s13040-015-0055-3) contains supplementary material, which is available to authorized users.

## Background

Advances in sequencing technology allow researchers to study microbial communities in new ways. Researchers use 16S rRNA sequencing to identify the bacteria present in microbial communities. These studies have found highly complex communities composed of hundreds or thousands of different bacteria types. Some microbial communities are found in or on other organisms. Known as microbiomes, these communities have been shown to play important roles in host health and disease. For example, in humans, gut microbiomes are important parts of digestion [1] and have been associated with obesity [2]. Microbial communities in the lungs may exacerbate cystic fibrosis [3].

The vagina microbiome is often composed of hundreds of different bacteria types, although only a few taxa may be at high abundance [4]. The composition of the vagina microbiome can be highly variable, both between women and through time [5]. Additionally the microbiome is associated with bacterial vaginosis (BV), but in ways that are not clear.

BV is a disease characterized by an overgrowth of certain microbe types in the vagina. It is highly prevalent, with estimates of affected women as high as almost 30 % [6]. Symptoms of BV include odor, discharge, and irritation. It is also associated with increased rates of preterm birth [7] and increased susceptibility to some STDs [8]. While no single microbial cause of BV has been found, the microbial community as a whole is associated with BV [9].

Researchers often use two main BV diagnostics. The Nugent score is a measure based on cell morphology that can range from 0 to 10, with a score of 7 or greater indicating BV [10]. The Amsel criteria include a vaginal pH greater than 4.5, a positive whiff test, the presence of clue cells, and the presence of discharge. The presence of three of these four criteria indicates BV [11].

Identifying the parts of the microbial community associated with BV is difficult. This is partly due to the large number of taxa found in the community and the even larger number of potential interactions between taxa. Variation in the microbial community between women and over time adds to the difficulty of the problem. Computational tools, however, may provide methods for studying these highly complex communities. In particular, machine learning methods may allow us to model complex relationships in the microbial community related to BV.

Machine learning methods are able to generate complex models describing the relationship between the microbial community and BV. Every machine learning method has a different technique for generating a classification model. However, the end result for each method is a model that classifies samples into BV categories. Two model characteristics are interesting. First, the model accuracy describes how well the model fits the data. Second, the important features of the model are those features that the model uses to classify the samples. These features allow the researcher to generate hypotheses about the underlying biology.

Previous research has found that classification models generated using genetic programming, random forests, and logistic regression classify microbial communities into BV categories with between 80 and 90 % accuracy [12]. This research identified two challenges to using machine learning classifiers to study microbial communities. First, when the classification models are deconstructed to determine which features are important to the model accuracy, each machine learning technique identifies different features. This makes it difficult to determine if the identified features are actually important, or if they are the result of technical artifacts. Additionally, it is difficult to distinguish between features that are critical to the accuracy of the classifier and features that are only marginally helpful. While an importance measure is calculated for each feature, this measure is often only effective in ranking features, rather than determining how much each feature adds to the overall accuracy.

In this study, we use subsets of the full feature set in order to address these problems. We add features sequentially to the classification models and observe how the accuracy changes. This allows us to determine how many features are necessary to obtain high classification accuracy. Additionally, we generate models using random feature subsets in order to obtain a feature importance measure that is consistent across machine learning techniques. We find that random forests and logistic regression classifiers identify largely similar microbial community features. However, it is not clear whether these methods improve upon simple correlations.

## Methods

We used datasets from studies published by Ravel *et al.* [4] and Srinivasan *et al.* [13]. The Srinivasan *et al.* dataset includes both Amsel BV and Nugent score BV, while the Ravel *et al.* dataset includes only Nugent score BV. The Nugent score is an integer value between zero and ten derived from the number of specific symptoms observed, with a score of seven to ten diagnosing the presence of BV, below four representing absence of BV, and intermediate scores being inconclusive for diagnosis. The Amsel diagnostic observes specific symptoms and diagnoses the presence or absence of BV from those observations. Both datasets contain patient symptom data. In particular, this made it possible for us to perform BV diagnostics for patients using Nugent scoring for data in the Ravel *et al.* dataset, even though they did not report Nugent scoring explicitly. In addition, both studies present the presence of menses and vaginal pH. The Srinivasan *et al.* study also reported extensive patient symptoms such as vaginal itching and vaginal discomfort. Both studies also present relative abundance data for OTUs identified by reference to standard databases using amplicons from 16S hypervariable regions (see papers for details). These OTUs were named by the closest taxonomic unit that matched them in standard databases, and by non-specific names (such as BVAB1, 2, and 3, which are uncharacterized clostridia-like bacteria) when necessary. Thus the input for our work includes patient symptoms, BV diagnostic data, and microbial community composition, which constitute the features for the learning algorithms we tested. This is a mixture of continuous parameters such as population relative abundance and pH and categorical variables such as Nugent score and symptoms.

The Ravel *et al.* study includes 396 asymptomatic women of whom 97 were BV+ using a Nugent score definition (Nugent score ≥7). The Srinivasan *et al.* study includes 220 women, of whom 97 were BV+ using Amsel criteria and 117 were BV+ using Nugent score. We processed the datasets using methods similar to that in [12], with the exception of not collapsing microbes into correlated groups.

We used two different machine learning algorithms to generate classification models, random forests (RF) and logistic regression (LR). The RF classifiers were implemented using the *randomForest* function in the R package randomForest [14]. We implemented LR classifiers using the *glmnet* function in the R package glmnet [15]. To identify important features of RF models, features were ranked according to their increase in node purity (INP). INP is a measure of how much each feature increases the classification accuracy of each decision tree, averaged across all trees in the ensemble. For LR, features were ranked by their mean coefficient magnitude in all cross-validation datasets divided by their standard deviation.

In addition to the RF and LR classifiers, we also calculated reliefF rankings and correlations between the features and BV. ReliefF is a feature selection algorithm that estimates the relevance of each feature by how well it separates similar samples into classes [16]. To calculate the reliefF rankings, we used the *attrEval* function in the R package CORElearn [17]. The Pearson correlation between each feature and BV was calculated using R’s *cor* function.

To prevent over fitting, we used ten fold cross validation. We split each dataset randomly into ten parts. We used nine of the parts to train the classification models and the remaining part to measure the model accuracy. We repeated this process using each of the ten parts as the test dataset.

For each of the cross validation datasets, we fit RF and LR models to the full feature set of the training data. We then calculated the importance of each feature to these models. ReliefF was used to generate a third feature ranking. We then used these rankings to select feature subsets in three different ways.

The first analysis selected the top N features from each of the feature rankings, where N ranged between two and 25. We refer to this analysis as “N feature” subsets below. The second analysis used a five-feature sliding window across each of the rankings. We refer to this analysis as “sliding window” subsets below. The third analysis selected the top 50 features from each ranking and combined them into a single list, from which we selected three thousand subsets of five features each at random. RF and LR classifiers were trained on each subset using the training data. The accuracy of each classifier was determined using the testing data. We refer to this analysis as “random features” below.

The classification accuracy for each model was measured using the area under the receiver-operator curve (AUC). The receiver-operator curve (ROC) describes the classifier accuracy in both BV positive and BV negative samples, thus representing both type 1 and type 2 error. The area under the ROC is often used as a summary of the model accuracy [18].

## Results and discussion

**Fig. 1:**
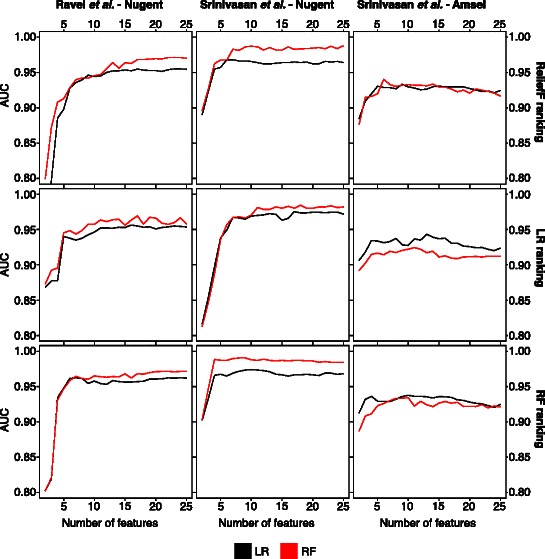
Top N feature subsets. This figure shows how the classification models perform as the number of features available to the models increases. Features are added according to their performance ranking using reliefF (top row), logistic regression (middle row), and random forests (bottom row). The model performance is measured using the area under the ROC (AUC). As can be seen, the top five features are often sufficient to obtain high classification accuracy

Top *N feature subsets* results help determine how accuracy improves with each feature addition. The features are added in order of perceived importance. If several features contribute additively and equally, a linear increase in accuracy would be expected. If only the top few features contribute substantially, the accuracy would reach its maximum quickly and then level off. More complex patterns may emerge if there are important interactions between features. Figure 1 shows the classification accuracy for RF and LR models as more features are added to the model. In every case, both RF and LR models classify samples with high accuracy after the inclusion of only a few features. With few exceptions, high accuracy is obtained with five or fewer features.

Differences in Amsel BV and Nugent score BV are apparent from these results. The classification accuracy is higher for Nugent score BV, indicating a better model fit. This may result from a closer link between Nugent score BV and the microbial community. It may also indicate that the relationship between Nugent score BV and the microbial community is more easily captured by the classification models. In other words, there may be a strong link between the microbial community and Amsel BV, but that link is complex and not fully exploited by the models. Alternatively, the Amsel BV classification may simply include more noise or error.

**Fig. 2:**
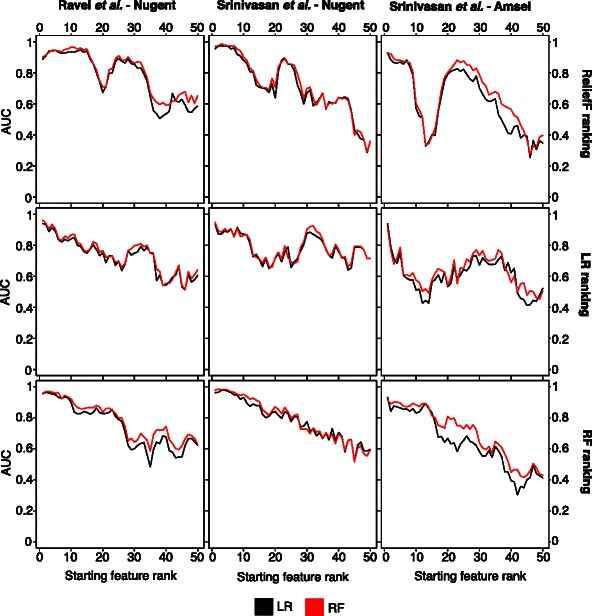
Five-feature sliding window subsets. This figure shows the accuracy of models using a sliding window of five features chosen consecutively from the ranked feature lists. Features are added according to their performance ranking using reliefF (top row), logistic regression (middle row), and random forests (bottom row). The model performance is measured using the area under the ROC (AUC)

*Sliding window subsets* results may show patterns that the top N features miss. For example, the first two features may individually be sufficient to obtain a high accuracy, in which case, the first feature in the top N subsets masks the relevance of the second feature. A sliding window makes it possible to determine how the features affect classification accuracy without the influence of the more important features of higher rank. Each successive window replaces the highest ranked feature in the previous window with the next lowest ranked feature. Figure 2 shows how the accuracy of RF and LR models changes as important features are replaced by lower ranked features.

The sliding window subsets for the reliefF and RF rankings show substantial stability in classification accuracy as lower ranked features replace the first few high ranked ones. This pattern appears reduced for the LR rankings. Additionally, the sliding window subsets for RF rankings generally show a consistent decrease in classification accuracy as the feature ranking decreases. The reliefF and LR rankings, however, show a more uneven decrease in accuracy with feature ranking. Similar patterns would be expected if the initial rankings were incorrect. While the reason for this poor performance is unknown, it may be partially due to sensitivity of the importance measures to sparse data.

**Fig. 3:**
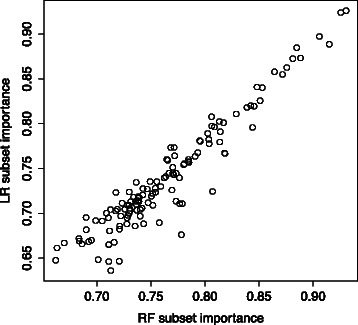
RF vs. LR feature subset importance. This figure compares the feature subset importance in RF and LR models in the Ravel *et al.* dataset. The feature subset importance values for RF classifiers are very similar to those for LR, especially for high ranked features

*Random subsets* results extend the sliding window analysis by removing its dependency on the initial feature ranking. This allows us to determine how each feature affects the model accuracy when combined with four other features. The size of the random group was chosen based on the top N analysis results. The inclusion of five features was often sufficient to produce models with accuracy as good as the full model. We calculated an importance measure for each feature by averaging the classification accuracies of all five-feature subset models containing the feature. This importance measure (referred to below as “subset importance”) can be calculated regardless of the model generating technique. The subset importance for the features is very similar for RF and LR, in contrast to previous results that found dissimilar rankings of important features [12]. Figure 3 compares the subset importance for RF and LR classifiers in the Ravel *et al.* dataset. Results for the Srinivasan *et al.* dataset are very similar and can be found in the supplemental information (Additional file 1).

Table 1 shows the top fifteen features for each dataset based on the mean classification accuracy across five feature subsets. Results from RF and LR classifiers were averaged together to produce the final ranking. The important features identified by the subset analysis are largely unsurprising. The microbial taxa that contribute substantially to the classification accuracy have been linked to BV in previous studies [13, 19, 20]. These taxa include *Gardnerella*, *Atopobium*, and *Eggerthella*. Various *Lactobacillus* species also rank highly.

**Table 1:** Top 15 important features. This table shows the top 15 features ranked by classification accuracy in five-feature subsets. The ranking shown here was obtained by averaging the results for the RF and LR classifiers

Ravel *et al.* Nugent	Srinivasan *et al.* Nugent	Srinivasan *et al.* Amsel
*Prevotella*	*Gardnerella vaginalis*	nugent
*Dialister*	pH	*Gardnerella vaginalis*
*Gardnerella*	*Atopobium vaginae*	*Eggerthella sp.* type 1
pH	clue	*Atopobium vaginae*
*Megasphaera*	*Eggerthella sp.* type 1	*Leptotrichia.amnionii*
*Atopobium*	*Dialister micraerophilus*	*Dialister micraerophilus*
*Eggerthella*	whiff	*Prevotella timonensis*
*Sneathia*	*Lactobacillus crispatus*	*Dialister sp.* type 2
*Peptoniphilus*	*Aerococcus christensenii*	*Lactobacillus crispatus*
*Parvimonas*	vag_fluid	*Parvimonas micra*
*Ruminococcaceae* 3	*Dialister sp.* type 2	*Aerococcus christensenii*
*L. crispatus*	*Prevotella timonensis*	BVAB2
*Aerococcus*	*Parvimonas micra*	*Megasphaera sp.* type 1
*Ruminococcaceae Incertae Sedis*	*Leptotrichia amnionii*	*Sneathia sanguinegens*
*L. iners*	*Megasphaera sp.* type 1	*Lactobacillus iners*

**Fig. 4:**
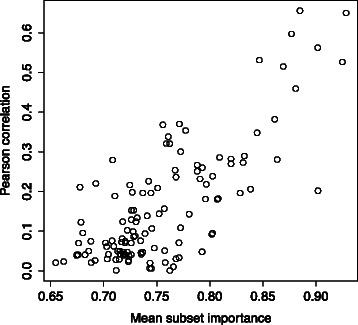
Feature subset importance vs. Pearson correlation. This figure shows the features plotted by the magnitude of their Pearson correlation with BV and their mean subset importance using both RF and LR classifiers for the Ravel *et al.* dataset. As can be seen, there is substantial similarity between the feature subset importance and the Pearson correlation

We next compared the subset importance measure to the Pearson correlation of each feature with BV. The results for the Ravel *et al.* dataset are shown in Fig. 4. The subset importance measure appears to rank features in a similar manner to the magnitude of the Pearson correlation. The Srinivasan *et al.* dataset shows similar patterns (Additional file 2).

The important feature rankings appear highly dependent on the importance measure used. While there is some overlap in the top five to ten features identified by each importance measure, there are many features ranked highly by one importance measure and not others (Additional file 3). It is not clear whether the ranking differences are due to noise or whether they may reflect some biological pattern.

## Conclusions

In this paper we found that only a few features are necessary to generate models with high BV classification accuracy. Additionally, there appears to be substantial redundancy in the microbial features. Random feature subsets allowed us to identify microbes important to BV classification. These taxa largely agree with those identified by other studies.

It is not clear if these classifiers find patterns that are any different from simple correlations. However, machine learning methods provide important accuracy measures that may help determine the number of features that are important. They may also indicate whether interaction terms are necessary to describe the system. Feature subset analysis illuminates many patterns and characteristics of the relationships between the microbial community and community characteristics such as BV. These methods may be generally useful for studying a wide range of microbial community related diseases and phenotypes.

## Additional files

Additional file 1 **RF vs. LR feature importance.** This figure compares the mean subset feature ranking for RF with that for LR in the Srinivasan *et al.* dataset. The feature ranking values for RF classifiers are very similar to those for LR classifiers. The Srinivasan *et al.* dataset using Nugent BV is shown on the left and the Srinivasan *et al.* dataset using Amsel BV is shown on the right. (PDF 84.4 KB)

Additional file 2 **Feature subset importance vs. Pearson correlation.** This figure compares the subset importance measure and the magnitude of the Pearson correlation. The Srinivasan *et al.* dataset using Nugent BV is shown on the left and the Srinivasan *et al.* dataset using Amsel BV is shown on the right. In both cases, the feature subset importance is similar to the Pearson correlation. (PDF 79.7 KB)

Additional file 3 **A comparison of the feature importance measures.** The black line is the magnitude of the Pearson correlation between the feature and BV. Two importance measures are shown for LR; the mean classification accuracy of random five-feature subsets and the mean coefficient magnitude across validation datasets divided by the standard deviation. Two importance measures are also shown for RF; the mean classification accuracy of random five-feature subsets and the increase in node purity (INP). All measures have been scaled to between 0 and 1 for comparison purposes except for the Pearson correlations. The datasets from the top are Ravel *et al.* Nugent BV, Srinivasan *et al.* Nugent BV, and Srinivasan *et al.* Amsel BV. The numbers represent the ranking of the feature using each importance measure. (PDF 55.7 KB)
