# Supplementary figures and images for: Machine learning classifiers provide insight into the relationship between microbial communities and bacterial vaginosis
Source: BioData Min. 2015 Aug 12;8:23. doi: 10.1186/s13040-015-0055-3 (PMC4542107; doi:10.1186/s13040-015-0055-3)

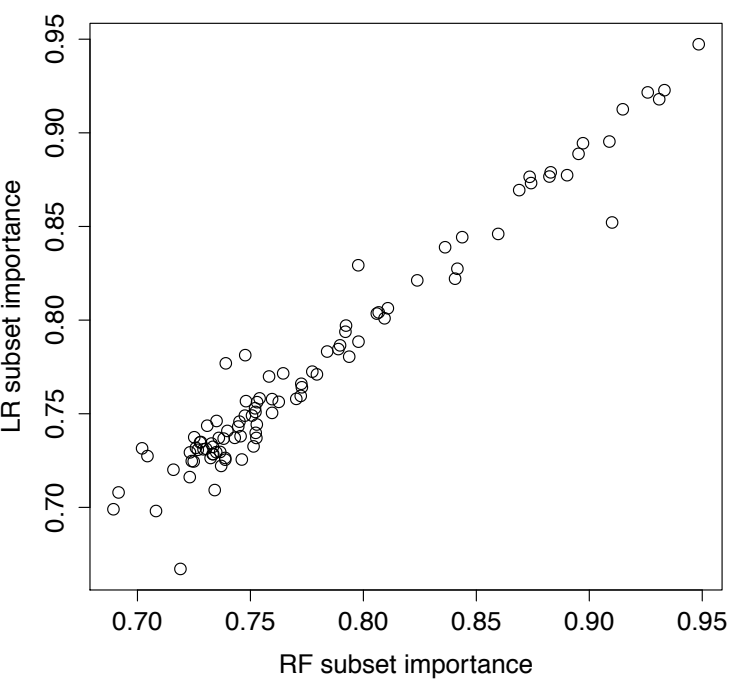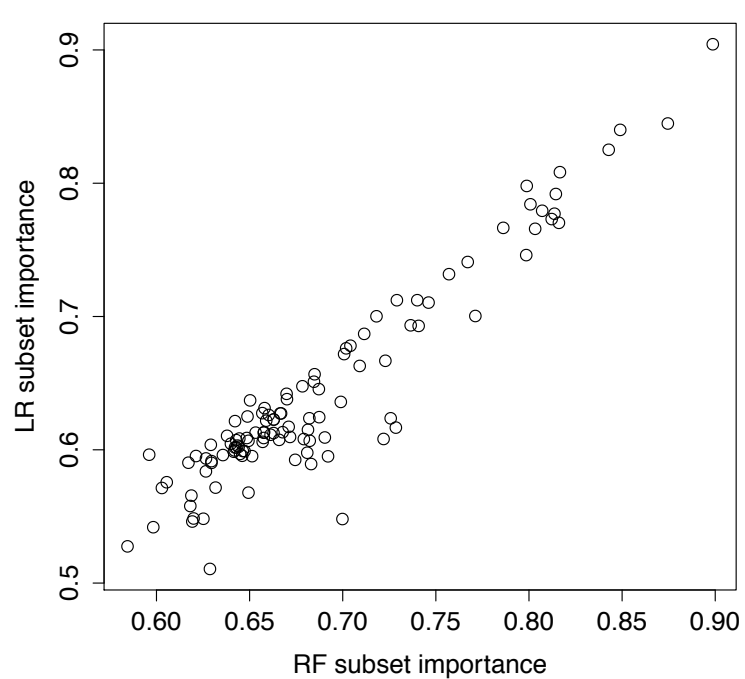

Supplement: Additional file 1 — RF vs. LR feature importance. This figure compares the mean subset feature ranking for RF with that for LR in the Srinivasan et al. dataset. The feature ranking values for RF classifiers are very similar to those for LR classifiers. The Srinivasan et al. dataset using Nugent BV is shown on the left and the Srinivasan et al. dataset using Amsel BV is shown on the right. (PDF 84.4 KB) [file 13040_2015_55_MOESM1_ESM.pdf]

Pearson correlation

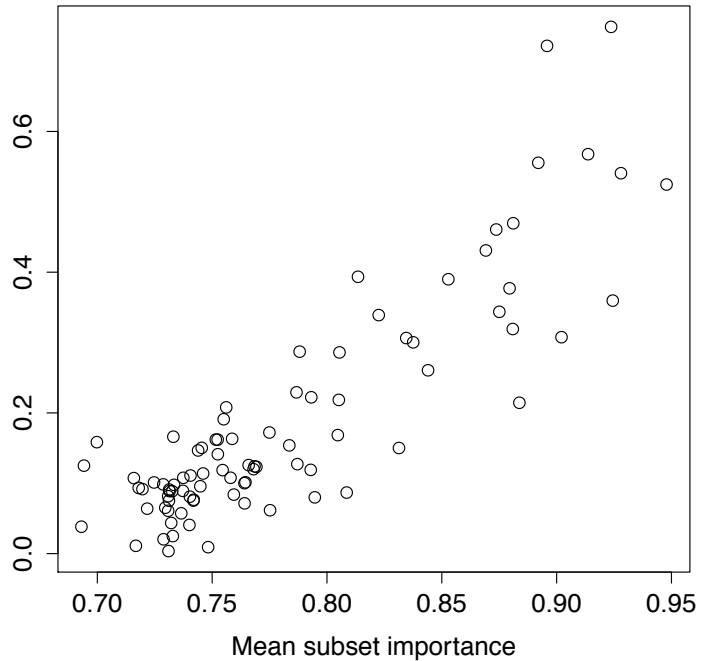

Pearson correlation

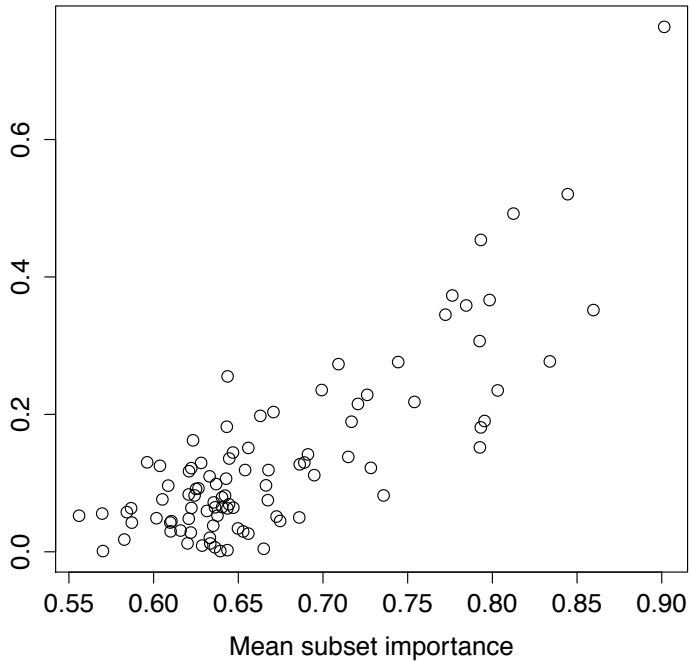

Supplement: Additional file 2 — Feature subset importance vs. Pearson correlation. This figure compares the subset importance measure and the magnitude of the Pearson correlation. The Srinivasan et al. dataset using Nugent BV is shown on the left and the Srinivasan et al. dataset using Amsel BV is shown on the right. In both cases, the feature subset importance is similar to the Pearson correlation. (PDF 79.7 KB) [file 13040_2015_55_MOESM2_ESM.pdf]

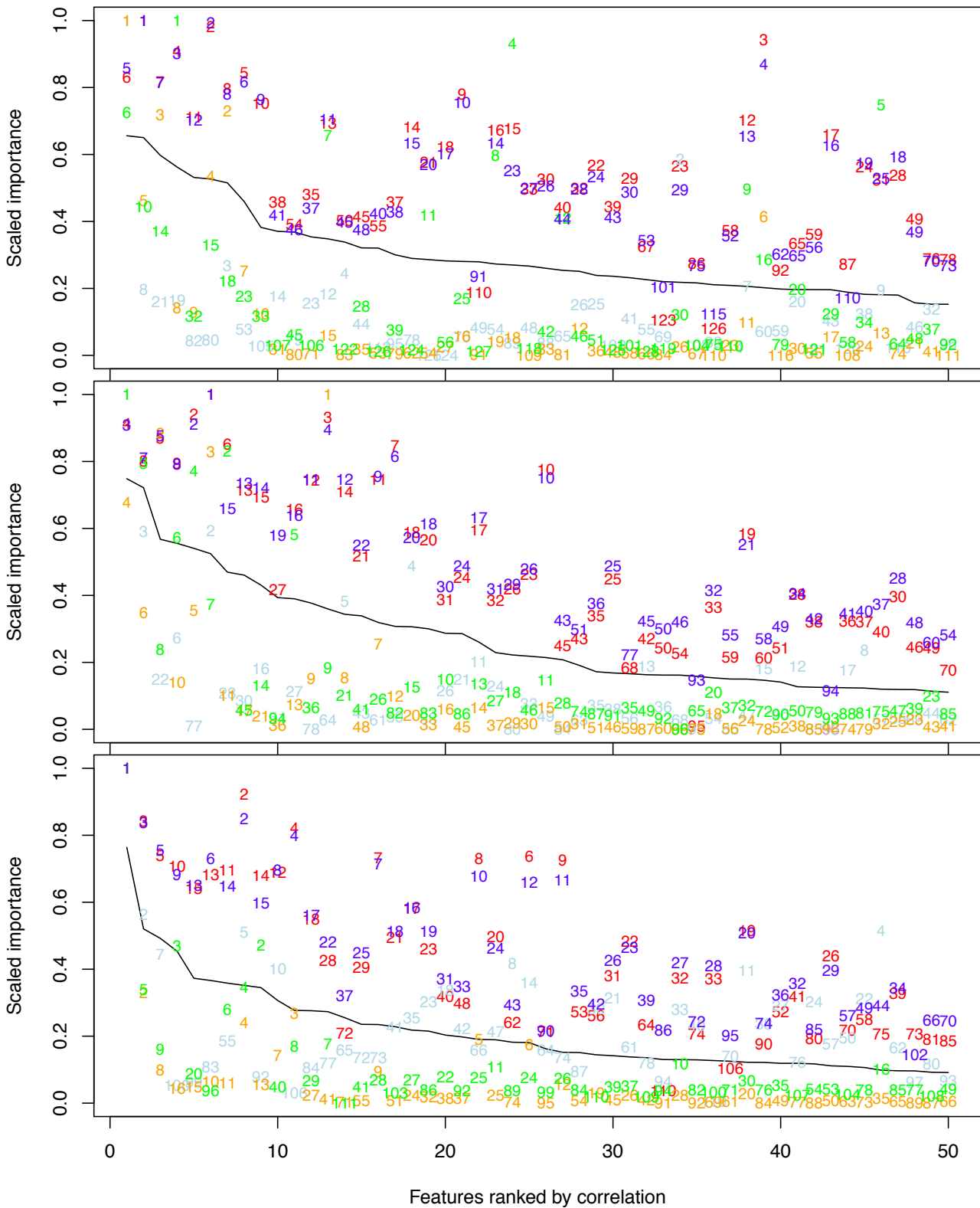

Supplement: Additional file 3 — A comparison of the feature importance measures. The black line is the magnitude of the Pearson correlation between the feature and BV. Two importance measures are shown for LR; the mean classification accuracy of random five-feature subsets and the mean coefficient magnitude across validation datasets divided by the standard deviation. Two importance measures are also shown for RF; the mean classification accuracy of random five-feature subsets and the increase in node purity (INP). All measures have been scaled to between 0 and 1 for comparison purposes except for the Pearson correlations. The datasets from the top are Ravel et al. Nugent BV, Srinivasan et al. Nugent BV, and Srinivasan et al. Amsel BV. The numbers represent the ranking of the feature using each importance measure. (PDF 55.7 KB) [file 13040_2015_55_MOESM3_ESM.pdf]
